# National HIV Testing Day and New Testing Recommendations

**Published:** 2014-06-27

June 27 marks the 20th annual observance of National HIV Testing Day, which promotes testing as an important first step in a strategy to detect, treat, and prevent human immunodeficiency virus (HIV) infection. HIV testing is entering a new era in the United States because of Food and Drug Administration approval of 1) combination tests that detect both HIV antigen and antibody, and 2) tests that accurately differentiate HIV-1 from HIV-2 antibodies. As a result, CDC has issued new guidelines, now available online, for HIV testing of serum or plasma specimens: *Laboratory Testing for the Diagnosis of HIV Infection: Updated Recommendations*.* Testing begins with a combination immunoassay that detects HIV-1 and HIV-2 antibodies and HIV-1 p24 antigen. All specimens reactive on this initial assay undergo supplemental testing with an immunoassay that differentiates HIV-1 from HIV-2 antibodies. Specimens that are reactive on the initial immunoassay and nonreactive or indeterminate on the antibody differentiation assay proceed to HIV-1 nucleic acid testing for resolution.

The updated recommendations allow detection of acute HIV infections that would be missed by antibody tests alone and can expedite entry of patients into care because of reduced turnaround time for test results. This issue of *MMWR* describes HIV screening programs in an urban health center in New York and an emergency department in New Orleans that used novel approaches to increase the number of patients screened for HIV. Both programs identified previously undiagnosed HIV infections. Use of the new testing algorithm allowed the New Orleans program to identify antibody-negative acute infections in five (6%) of the 77 patients with newly diagnosed HIV.

Additional information on HIV testing for health professionals and the public is available at http://www.cdc.gov/hiv/testing.

## Footnotes

* Available at http://www.cdc.gov/hiv/testing/lab/guidelines.

